# Sex-Specific Outcome Following Targeted Blood Pressure, Oxygenation, and Fever Control After Out-of-Hospital Cardiac Arrest

**DOI:** 10.1016/j.jacadv.2025.102056

**Published:** 2025-08-20

**Authors:** Sarah L.D. Holle, Martin A.S. Meyer, Jacob E. Møller, Jesper Kjærgaard, Henrik Schmidt, Simon Mølstrøm, Johannes Grand, Laust E.R. Obling, Helle Søholm, Martin Frydland, Christian Hassager

**Affiliations:** a Department of Cardiology, The Heart Centre, Copenhagen University Hospital, Rigshospitalet, Copenhagen, Denmark; b Department of Cardiology, Odense University Hospital, Odense, Denmark; c Department of Cardiothoracic Anaesthesia, Odense University Hospital, Odense, Denmark; d Department of Cardiology, Skånes University Hospital Lund, Lund, Sweden; e The Faculty of Health and Medical Sciences, University of Copenhagen, Copenhagen, Denmark

**Keywords:** mean arterial pressure, mortality, out-of-hospital cardiac arrest, oxygen, sex differences, temperature control

## Abstract

**Background:**

Previous studies show higher mortality for female patients with out-of-hospital cardiac arrest (OHCA) compared to males. The BOX (Blood Pressure and Oxygenation Targets in Post Resuscitation Care) trial investigated the effects of different mean arterial pressure (MAP) targets, oxygenation levels, and durations of fever control, finding no significant differences between groups.

**Objectives:**

The purpose of this study was to explore the association between sex and mortality rates by examining both the individual and possible interactive effects of the interventions in the BOX trial for both sexes.

**Methods:**

This two-center, randomized trial included adult comatose OHCA patients (age ≥18 years) of presumed cardiac cause. Participants were assigned to a blinded MAP target of 63 or 77 mm Hg, open-label arterial oxygen levels of 9–10 or 13–14 kPa, and fever prevention for 36 or 72 hours. The primary outcome was 1-year all-cause mortality.

**Results:**

Of 789 comatose OHCA patients, 152 (19%) were females. The median ages of females and males were similar, 64 (51-71) years and 64 (55-73) years, respectively. Comorbidities and characteristics of the cardiac arrest were comparable between sexes except for ischemic heart disease (females: 12%, males 24%). Mortality in females was 42% and 35% in males; HR: 1.27 (95% CI: 0.96-1.69). None of the targeted interventions had a statistically significant impact on mortality for either sex. No mortality difference between sexes was observed across the interventions.

**Conclusions:**

Among comatose patients following OHCA, no differences were observed between sexes in 1-year mortality or the efficacy of the blood pressure, oxygen, or temperature intervention.

Out-of-hospital cardiac arrest (OHCA) is one of the leading causes of death (Central Illustration).^1^ Previous studies have demonstrated that females experiencing OHCA exhibit lower survival rates compared to males.^2^, ^3^, ^4^ This sex disparity has been attributed to several factors, including differences in the pathophysiology of cardiac events, hormonal influences, and variations in treatment including postresuscitation.^2^^,^^5^ Notably, female OHCA patients tend to be older and less likely to have shockable initial rhythm compared to males, both of which are strong predictors of poor outcome following resuscitated cardiac arrest.^6^^,^^7^

**Central Illustration:**
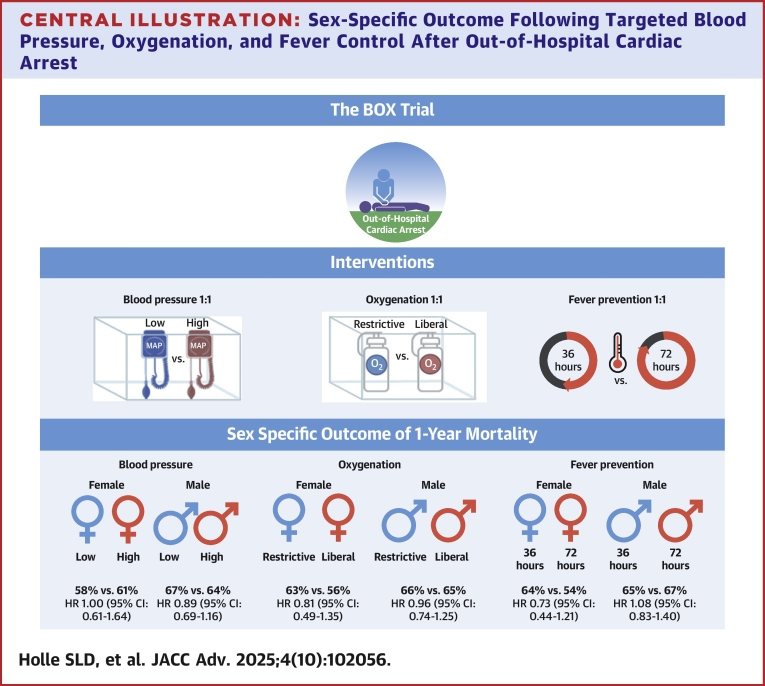
**Sex-Specific Outcome Following Targeted Blood Pressure, Oxygenation, and Fever Control After Out-of-Hospital Cardiac Arrest** Overview of sex-specific outcome in the BOX trial following out of-hospital cardiac arrest. Patients were assigned to MAP targets of 63 or 77 mm Hg, arterial oxygen levels of 9 to 10 or 13 to 14 kPa, and temperature control durations of 36 or 72 hours. No significant sex-based differences in 1-year mortality or treatment effects were observed. Abbreviation as in Figure 1.

The recent BOX (Blood Pressure and Oxygenation Targets in Post Resuscitation Care) trial assessed the influence of controlled oxygenation via mechanical ventilation,^8^ different mean arterial pressure (MAP) levels,^9^ and different temperature control duration^10^ on the prognosis of comatose OHCA patients. These studies^8^, ^9^, ^10^ aimed to optimize postresuscitation management by exploring whether the different targets could influence neurological recovery and overall survival. No significant differences in outcomes between any of the target groups were found, suggesting that other factors may play a more critical role in determining patient prognosis.

Studies have shown conflicting results regarding sex disparities in unfavorable outcomes after OHCA.^2^^,^^3^^,^^6^^,^^11^, ^12^, ^13^ These inconsistencies may be due to underrepresentation of females in studies, differences in study inclusion criteria, treatment approaches, or adjustment for known risk factors. We therefore investigated the influence of sex on post-OHCA outcomes of patients enrolled in the BOX trial and the treatment effects according to allocated oxygen target, MAP levels, and duration of temperature control. Understanding these potential differences could be crucial for developing tailored treatments that may improve survival for OHCA patients.

The objective of this study is to investigate the association between sex and 1-year mortality by analyzing the individual and combined effects of targeted blood pressure, oxygenation, and temperature control in comatose OHCA patients.

## Methods

### Trial design

This is a post hoc substudy from the BOX trial—an investigator-initiated, dual-center, open-label, 2-by-2-by-2 factorial, randomized trial. The BOX trial was conducted from March 2017 to December 2021 at 2 Danish tertiary heart centers. The BOX trial aimed to evaluate the effects of different oxygenation strategies, blood pressure targets, temperature control targets on comatose patients resuscitated after OHCA of presumed cardiac cause.

The Regional Ethics Committee of the Capital Region of Denmark approved the trial. The handling of patient data was approved by the Danish Data Protection Agency. The BOX trial is registered at ClinicalTrials.gov, NCT03141099. The method of the study has previously been described,^14^ and the primary findings of the trial as well as 1-year mortality have previously been published.^8^, ^9^, ^10^^,^^15^ The 1-year mortality was assessed by means of the electronic medical record which is linked to the national person data registry in Denmark.

### Participants

Eligible patients were adults (≥18 years) who had been resuscitated after an OHCA with a presumed cardiac cause, achieved sustained return of spontaneous circulation (ROSC) for ≥20 minutes, and remained comatose at hospital arrival. Patient inclusion had to occur within 240 minutes of ROSC. Key exclusion criteria included unwitnessed asystole and suspected acute intracranial bleeding or stroke. Included patients were divided into female and male defined as the biological sex assigned at birth.^16^

### Interventions

Patients were randomly (1:1) assigned to the following 3 interventions independent of one another: 1) an open-label partial pressures of arterial oxygen target of 9 to 10 kPa (68-75 mm Hg) for the restrictive-target group or 13 to 14 kPa (98-105 mm Hg) for the liberal-target group; 2) a blinded target for MAP set at either 63 mm Hg or 77 mm Hg; and 3) an open-label device-based fever prevention was administered as temperature control: 24 hours at 36 °C, followed by 12 or 48 hours at 37 °C. Patients would receive a total of either 36 or 72 hours of temperature control, depending on the assigned intervention arm. The interventions have been described in detail previously.^8^, ^9^, ^10^ Patients were randomized as soon as possible after hospital admission.

All patients received initial treatment in an intensive care unit, adhering to the European Resuscitation Council guidelines that were in effect during the trial’s design and execution.^17^ As part of standard care, all patients were analog-sedated and received mechanical ventilation.

### Outcomes

The primary outcome was 1-year all-cause mortality for both sexes in the treatment groups—comparing low to high blood pressure, liberal to restrictive oxygenation, and shorter to longer durations of temperature control. One secondary outcome was the potential interactive effect of the 3 interventions on sex-specific 1-year mortality. Other secondary outcomes were neurological outcomes and serious adverse events (SAEs) for both sexes in the treatment groups. The neurological outcomes were defined as Cerebral Performance Category, modified Rankin Scale, Montreal Cognitive Assessment, and neuron-specific enolase level.

The Cerebral Performance Category scale ranged from 1 to 5, with 1 indicating no symptoms and 5 representing death. A score of 3 or 4 on this scale reflected a state of severe disability, coma, or a persistent vegetative state. The modified Rankin Scale was used to assess the degree of disability or dependence in daily activities, ranging from 0 to 6. A score of 0 indicated no symptoms, 1 signified no clinically significant disability, 2 slight disability, 3 moderate disability, 4 moderately severe disability, 5 severe disability, and 6 corresponded to death. The Montreal Cognitive Assessment score was used to evaluate cognitive function, yielding scores from 0 to 30. A score of ≥26 was considered within the normal range.

### Statistics

Continuous outcomes were reported as means with SDs or medians with IQRs. The 1-year mortality outcome was analyzed using a Cox proportional hazards model, adjusted for site, across all 3 interventions in alignment with primary publications and outcome of 1-year mortality from the BOX trial.^8^, ^9^, ^10^^,^^15^

Results were reported as HRs with 95% CIs and *P* values. The proportional hazards assumption has been assessed in the main BOX trial publications, and no violations were found.^8^, ^9^, ^10^ Additionally, for both sex potential interactions between the interventions were examined in Cox proportional hazards models, also adjusted for site.

## Results

Among 789 enrolled patients, 152 (19%) were females. The ages of females and males were comparable, at 64 (51-71) years and 64 (55-73) years, respectively. The patients' comorbidities and characteristics of cardiac arrest were similar between the sexes, except for a history of ischemic heart disease, which was more frequently observed in males (24% vs 12%) (Table 1).

**Table 1:** Baseline Characteristics of the Patients

	Females (n = 152)	Males (n = 637)
Age (y), median (IQR)	64 (51-71)	64 (55-73)
Comorbidities, n (%)		
Hypertension	75 (50)	287 (45)
Diabetes	19 (13)	91 (14)
IHD	18 (12)	154 (24)
Chronic heart failure	22 (14)	115 (18)
COPD	15 (10)	48 (8)
Prior stroke	13 (9)	46 (7)
Chronic kidney disease	10 (7)	29 (5)
Characteristics of the cardiac arrest		
Shockable rhythm, n (%)	133 (88)	573 (90)
Witnessed arrest, n (%)	136 (89)	536 (84)
Bystander cardiopulmonary resuscitation, n (%)	127 (85)	552 (88)
Time to ROSC (min), median (IQR)	18 (12-27)	18 (12-25)
Findings and procedures at hospital arrival		
pH, median (IQR)	7.23 (7.14-7.28)	7.24 (7.15-7.30)
Lactate (mmol/L), median (IQR)	5.2 (3.3-8.0)	4.9 (2.8-7.9)
Mean arterial blood pressure (mm Hg), median (IQR)	82 (72-94)	75 (67-85)
PaO_2_ (kPa), median (IQR)	9.3 (6.5-19.7)	10.8 (6.9-19.4)
Temperature (^o^C), median (IQR)	35.4 (34.4-36.0)	35.5 (34.8-36.1)
ST-segment elevation ECG, n (%)	66 (44)	284 (46)
Left ventricular ejection fraction (%), median (IQR)	35 (25-50)	35 (25-45)
Vasoactive-inotropic score in the CICU, median (IQR)	0.00 (0.00-10.0)	0.10 (0.00-10.00)
Immediate coronary angiography, n (%)	137 (90)	585 (92)
Percutaneous coronary intervention, n (%)^a^	63 (46)	273 (47)

CICU = cardiac intensive care unit; COPD = chronic obstructive pulmonary disease; ECG = electrocardiogram; IHD = ischemic heart disease; PaO_2_ = partial pressures of arterial oxygen; ROSC = return of spontaneous circulation.

a The percentage represents the number of percutaneous coronary interventions performed as a proportion of the total immediate coronary angiographies.

**Figure 1:**
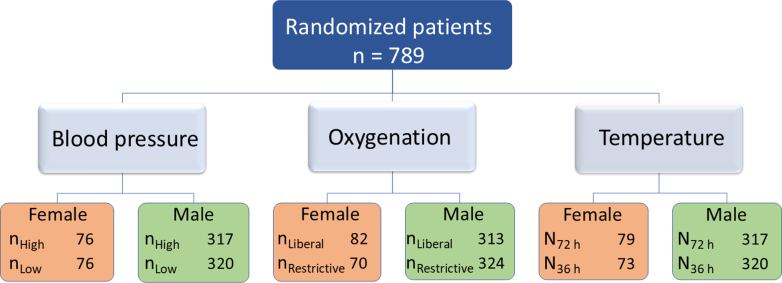
**Distribution of Participants and Sex Across the 3 Interventions in the BOX Trial** BOX = Blood Pressure and Oxygenation Targets in Post Resuscitation Care.

The patients were randomized across 3 different interventions with equal representation of females in each group (Figure 1). For the oxygenation intervention, 82 females and 312 males were allocated to a liberal oxygenation target, and 70 females and 324 males to a restrictive oxygenation target. For the blood pressure intervention, 76 females and 317 males were randomized to a high MAP target, and 76 females and 320 males were randomized to a low MAP target. In the device-based fever prevention intervention, 79 females and 316 males were assigned to 72 hours of temperature control, and 73 females and 320 males were assigned to 36 hours of temperature control. Baseline characteristics across all 3 interventions are presented in Supplemental Tables 1 to 3. The baseline characteristics between the 2 strategies for all 3 interventions were similar for males and females. Except for females allocated to the high blood pressure target were more likely to have a history of ischemic heart disease prior to admission compared to those in the low blood pressure target group (17% vs 7%).

Follow-up was complete for all survivors except 2 survivors at 1 year; hence, complete 1-year follow-up was available for 787 of the 789 patients.

**Figure 2:**
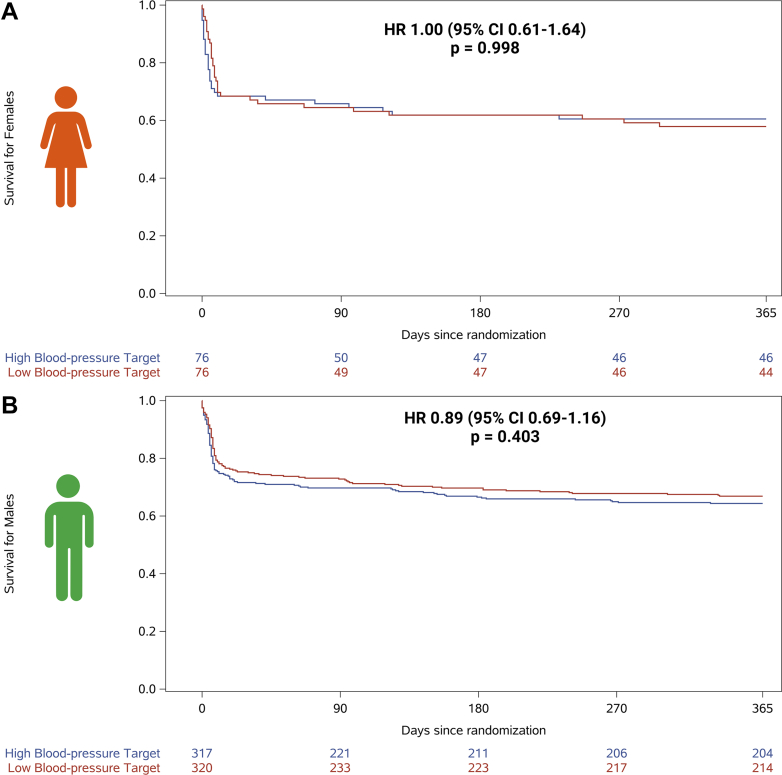

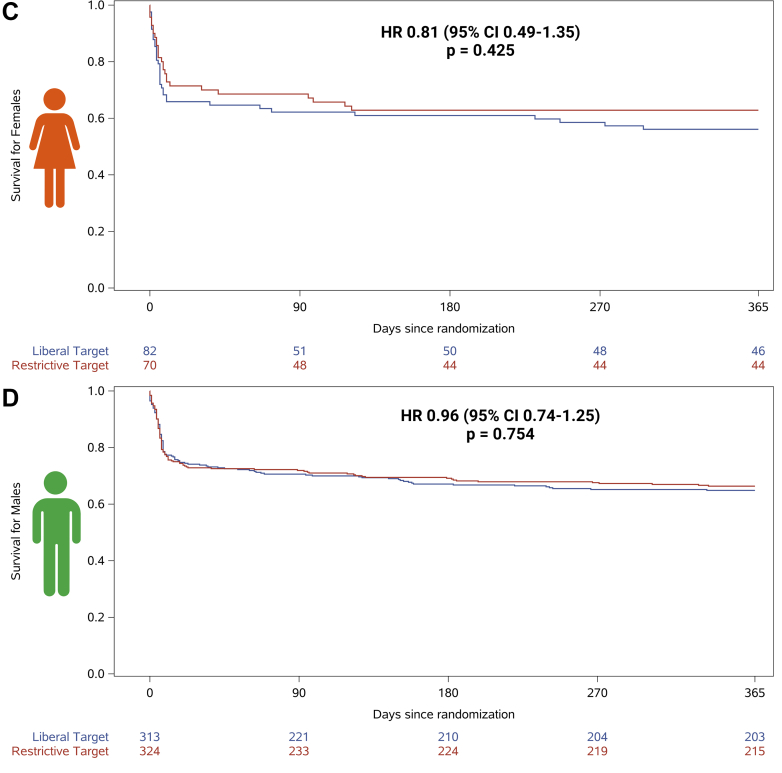

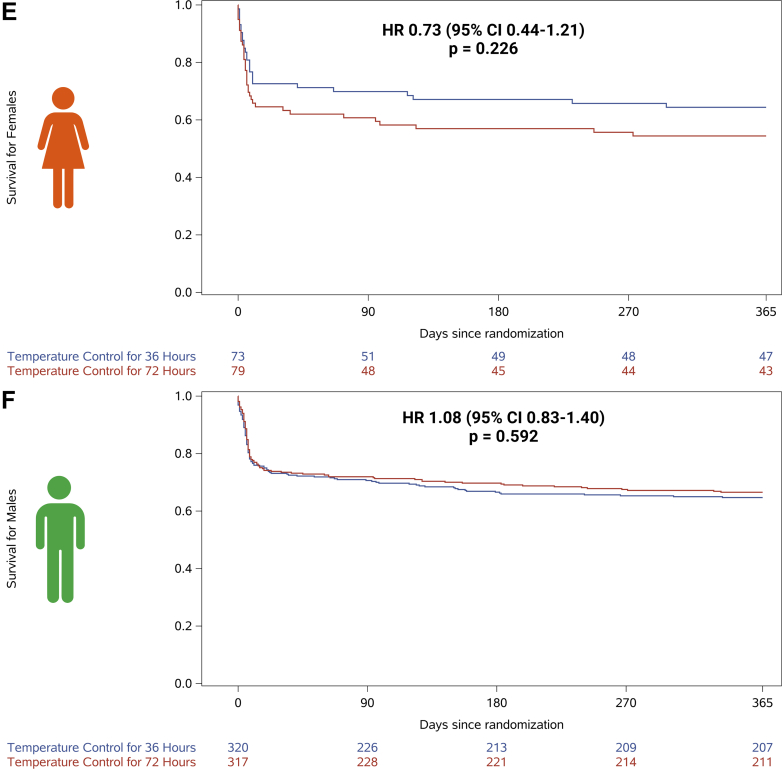
Comparison Across All 3 Interventions (Blood Pressure, Oxygenation, and Temperature Control) Effect on 1-Year Survival After Out-of-Hospital Cardiac Arrest by Sex (A) 1-year survival of females allocated to restrictive compared to liberal oxygenation targets, (B) 1-year survival of males allocated to restrictive compared to liberal oxygenation targets, (C) 1-year survival of females allocated to low compared to high blood pressure targets, (D) 1-year survival of males allocated to low compared to high blood pressure targets, (E) 1-year survival of females allocated to fever prevention for 36 compared to 72 hours, and (F) 1-year survival of males allocated to fever prevention for 36 compared to 72 hours.

For the both sexes, no differences in 1-year all-cause mortality were observed when comparing low vs high blood pressure target (female: 58% vs 61%; HR: 1.00 [95% CI: 0.61-1.64] and male: 67% vs 64%; HR: 0.89 [95% CI: 0.69-1.16], interaction for sex *P* = 0.09) (Figures 2A and 2B and Supplemental Table 4). No differences in neurological outcomes and SAEs were observed when comparing the blood pressure targets, except for metabolic disorder, which was more frequent among females receiving the low blood pressure target (14% vs 4%) (Table 2). When comparing restrictive vs liberal oxygenation targets, no sex-specific treatment effect on 1-year mortality was observed (females: 63% vs 56%; HR: 0.81 [95% CI: 0.49-1.35]; males: 66% vs 65%; HR: 0.96 [95% CI: 0.74-1.25], interaction for sex *P* = 0.10) (Figures 2C and 2D). No differences in neurological outcomes and SAEs were observed when comparing the oxygenation targets, except for acute kidney injury with renal replacement, which was more frequent among females receiving the liberal oxygenation target (16% vs 1%) (Table 2). Temperature control for shorter vs longer durations did not show a significant treatment effect on 1-year mortality for either sex (females: 64% vs 54%; HR: 0.73 [95% CI: 0.44-1.21]; males: 65% vs 67%; HR: 1.08 [95% CI: 0.83-1.40], interaction for sex *P* = 0.09) (Figures 2E and 2F). No differences in neurological outcomes and SAEs were observed when comparing the duration of temperature control, except for metabolic disorder, which was more frequent among females receiving the shorter duration of temperature control (15% vs 4%) (Table 2). After adjustment for the other intervention targets, these results of 1-year all-cause mortality across each intervention target remained nonsignificant for both sexes (Table 3).

**Table 2:** Outcome and Adverse Events for Each Intervention

Blood Pressure	Female		Male	
	High (n = 76)	Low (n = 76)	High (n = 317)	Low (n = 320)
CPC score at 1 year, median (IQR)	1 (1-5)	2 (1-5)	1 (1-5)	1 (1-5)
Modified Rankin Scale score at 1 year, median (IQR)	2 (0-6)	2 (1-6)	1 (0-6)	1 (0-6)
MoCA score at day 90, median (IQR)	27 (23-28)	25.5 (23-29)	27 (24-29)	27 (24-29)
Neuron-specific enolase level at 48 h (μg/L), median (IQR)	17.2 (10.9-26.9)	23.9 (12.9-40.4)	18.2 (10.4-38.9)	16.9 (10.5-31.4)
Serious adverse events				
Infection, n (%)^a^	16 (21)	23 (30)	86 (27)	87 (27)
Arrhythmia, n (%)^b^	10 (13)	9 (12)	49 (15)	41 (13)
Any bleeding, n (%)^c^	16 (21)	21 (28)	66 (21)	71 (22)
Uncontrolled bleeding, n (%)^c^	4 (5)	5 (7)	18 (6)	11 (3)
Acute kidney injury with renal- replacement, n (%)	8 (11)	6 (8)	33 (10)	34 (11)
Electrolyte disorder, n (%)^d^	4 (5)	10 (13)	19 (6)	24 (8)
Metabolic disorder, n (%)^e^	3 (4)	11 (14)	28 (9)	20 (6)
Seizure, n (%)^f^	13 (17)	18 (24)	63 (20)	70 (22)

**Oxygenation**	**Liberal (n = 82)**	**Restrictive (n = 70)**	**Liberal (n = 313)**	**Restrictive (n = 324)**

CPC score at 1 year, median (IQR)	2 (1-5)	1 (1-5)	1 (1-5)	1 (1-5)
Modified Rankin Scale score at 1 year, median (IQR)	2 (1-6)	2 (1-6)	1 (0-6)	1 (0-6)
MoCA score at day 90, median (IQR)	26.5 (23-28)	25 (23-28)	26.5 (24-29)	27 (24-28)
Neuron-specific enolase level at 48 h (μg/L), median (IQR)	20.6 (13.2-33.9)	19.3 (10.7-39.5)	17.2 (10.1-36.4)	18.3 (11.0-33.7)
Serious adverse events				
Infection, n (%)^a^	19 (23)	20 (29)	90 (29)	83 (26)
Arrhythmia, n (%)^b^	10 (12)	9 (13)	42 (13)	48 (15)
Any bleeding, n (%)^c^	22 (27)	15 (21)	70 (22)	67 (21)
Uncontrolled bleeding, n (%)^c^	7 (9)	2 (3)	14 (4)	15 (5)
Acute kidney injury with renal- replacement, n (%)	13 (16)	1 (1)	34 (11)	33 (10)
Electrolyte disorder, n (%)^d^	6 (7)	8 (11)	19 (6)	24 (7)
Metabolic disorder, n (%)^e^	7 (9)	7 (10)	21 (7)	27 (8)
Seizure, n (%)^f^	20 (24)	11 (16)	63 (20)	70 (22)

**Temperature Control**	**36 h (n = 73)**	**72 h (n = 79)**	**36 h (n = 320)**	**72 h (n = 317)**

CPC score at 1 year, median (IQR)	1 (1-5)	2 (1-5)	1 (1-5)	1 (1-5)
Modified Rankin Scale score at 1 year, median (IQR)	2 (0.5-6)	2.5 (1-6)	1 (0-6)	1 (0-6)
MoCA score at day 90, median (IQR)	26 (23-28)	26 (23-29)	26 (24-29)	27 (24-28)
Neuron-specific enolase level at 48 h (μg/L), median (IQR)	18.2 (10.4-35.9)	21.2 (12.7-38.4)	19.1 (11.2-34.2)	16.4 (10.1-36.7)
Serious adverse events				
Infection, n (%)^a^	23 (32)	16 (20)	79 (25)	94 (30)
Arrhythmia, n (%)^b^	10 (14)	9 (11)	52 (16)	38 (12)
Any bleeding, n (%)^c^	18 (25)	19 (24)	66 (21)	71 (22)
Uncontrolled bleeding, n (%)^c^	2 (3)	7 (9)	15 (5)	14 (4)
Acute kidney injury with renal- replacement, n (%)	6 (8)	8 (10)	33 (10)	34 (11)
Electrolyte disorder, n (%)^d^	9 (12)	5 (6)	21 (7)	22 (7)
Metabolic disorder, n (%)^e^	11 (15)	3 (4)	23 (7)	25 (8)
Seizure, n (%)^f^	12 (16)	19 (24)	72 (23)	61 (19)

CPC = Cerebral Performance Category; MoCA = Montreal Cognitive Assessment score.

a Infection: severe sepsis, septic shock, pneumonia during or after ventilator therapy, and other.

b Arrhythmia: ventricular fibrillation, ventricular tachycardia, tachycardia (>130 beats/min), bradycardia (<40 beats/min), atrial flutter, atrial fibrillation, need for pacing, or circulatory collapse mandating cardiopulmonary resuscitation.

c Bleeding: Any bleeding included uncontrolled bleeding (>1 unit of blood per 10 kg of body weight per hour), bleeding causing death, or symptomatic bleeding in a critical organ (eg, intracranial, intraspinal, intraocular, intraarticular, or pericardial bleeding).

d Electrolyte: hypokalemia (potassium level <3.0 mmol/L), hypophosphatemia (phosphate level <0.7 mmol/L), or hypomagnesemia (magnesium level <0.7 mmol/L).

e Metabolic disorders: sustained hyperglycemia (blood glucose level >10 mmol/L for >4 hours) or hypoglycemia (blood glucose level <3.0 mmol/L for >4 hours).

f Seizures: tonic–clonic, myoclonic, and electrographic status epilepticus.

**Table 3:** Cox Proportional Hazard Models for 1-Year All-Cause Mortality for Each Intervention Adjusted for Site and the Other Two Intervention Strategies

Intervention Strategies^a^	HR (95% CI)	*P* Value
Blood pressure, female (HR with low)	0.99 (0.60-1.63)	0.976
Blood pressure, male (HR with low)	0.89 (0.68-1.16)	0.390
Oxygenation, female (HR with restrictive)	0.80 (0.48-1.33)	0.389
Oxygenation, male (HR with restrictive)	0.96 (0.74-1.25)	0.764
Temperature control, female (HR with 36 h)	0.72 (0.43-1.20)	0.210
Temperature control, male (HR with 36 h)	1.08 (0.83-1.41)	0.571

a Comparison of intervention strategies: For the blood pressure intervention, a target mean arterial pressure of 63 mm Hg was compared to 77 mm Hg for both sexes. For the oxygenation intervention, an arterial oxygen concentration range of 9 to 10 kPa was compared to 13 to 14 kPa for both sexes. For the temperature control intervention, device-based fever prevention was implemented for 36 hours and compared to 72 hours for both sexes.

**Figure 3:**
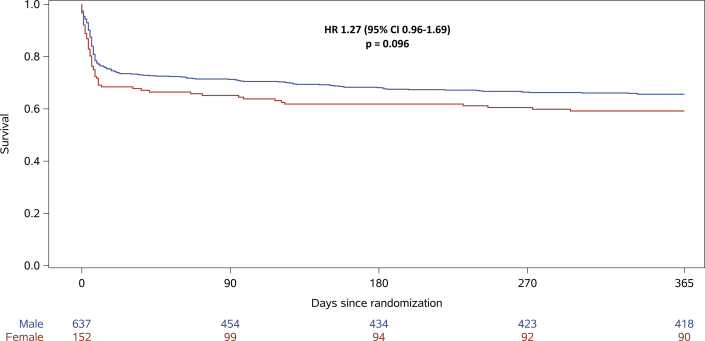
Sex Differences in 1-Year Survival After Out-of-Hospital Cardiac Arrest

At 1 year, 64 of 152 females (42%) and 224 of 637 males (35%) died, HR: 1.27 (95% CI: 0.96-1.69), *P* = 0.092, with adjustment for site (Figure 3). This result remained unaltered after adjustment for all 3 treatment interventions (HR: 1.27 [95% CI: 0.96-1.69], *P* = 0.096).

## Discussion

The present study is, to the best of our knowledge, the first to investigate sex-specific outcomes in a randomized setting following the targeting of blood pressure, oxygenation, and fever prevention in comatose OHCA patients. This substudy from the BOX trial found no interaction between sex and any of the 3 treatment interventions (low compared to high MAP, liberal compared to restrictive oxygenation, or shorter compared to longer duration of temperature control).

Targeted blood pressure in OHCA patients has been examined for its potential benefits on outcome by ensuring sufficient perfusion.^9^^,^^18^^,^^19^ Notably, the BOX trial reported no significant differences in 1-year mortality between higher and lower MAP targets. But a higher frequency of metabolic disorders was observed among females receiving lower MAP level. It is worth considering that some patients may benefit from individualized MAP targets. Female patients were underrepresented in the BOX trial, which could have diluted the treatment effect. Two additional smaller trials also indicated no difference in 30- and 180-day mortality between different MAP levels, with a similar underrepresentation of females.^18^^,^^19^ Our study corroborates these findings, showing no significant difference in 1-year mortality between MAP levels of 63 mm Hg and 77 mm Hg among male and female patients.

Optimal oxygenation levels have been considered crucial for patient outcomes, as hypoxic-ischemic brain injury is the leading cause of death in patients who have been resuscitated after cardiac arrest. However, high fraction of oxygen might induce reperfusion injury.^20^^,^^21^ Previous small-scale studies, which were not powered to detect mortality differences and included few female participants, have shown neutral results.^21^ Our study found no significant difference in 1-year mortality between restrictive and liberal oxygenation strategies for both male and female patients. However, a higher frequency of acute kidney injury with renal replacement was observed among females receiving liberal oxygenation.

Preventing fever following cardiac arrest aims to mitigate the effects of hypoperfusion, hypoxia, and subsequent possible reperfusion injury.^22^ However, a randomized trial has demonstrated no mortality difference between temperature controls set at 33 °C and 36 °C, including analyses stratified by sex.^23^ This study supports these previous findings, indicating no significant difference in 1-year mortality between 36 and 72 hours of temperature control for both male and female patients. But females receiving shorter temperature control showed a higher frequency of metabolic disorder.

This study demonstrated no significant differences in 1-year all-cause mortality rates between males and females. Previous register-based studies suggested lower survival rates among females, often due to older age at arrest and fewer shockable rhythms.^12^^,^^24^^,^^25^ Our randomized study included hospital-admitted OHCA patients who achieved ROSC and exhibited favorable characteristics allowing for hospital admission. The cohort was younger than typical prehospital OHCA cohorts,^2^^,^^12^ with similar ages between sexes, reducing age-related confounders. We observed no difference in bystander cardiopulmonary resuscitation, time to first defibrillation, or percutaneous coronary intervention, unlike other studies reporting treatment disparities.^12^^,^^24^^,^^25^

This study is a selected cohort of OHCA patients primarily caused by presumed cardiac causes. The similar risk profiles and care between sexes in this study may explain the absence of disparities in 1-year all-cause mortality. These findings align with a meta-analysis which found that female sex was not associated with increased mortality,^13^ and other studies indicating any initial unfavorable outcomes for females diminish after adjusting for risk factors.^2^^,^^11^^,^^12^ The findings from this study suggest that, although sex disparities in OHCA outcomes have been documented in previous literature, these differences may be less pronounced when risk factors and treatment between sexes are comparable.

There are biological differences between males and females that could play a role in how each sex responds to the treatment. Sex hormones have been found to be associated with mortality in OHCA patients.^26^ Our study found no differences in 1-year all-cause mortality rates between the sexes were found in any of the 3 treatment groups—whether comparing low to high blood pressure, liberal to restrictive oxygenation, or shorter to longer durations of temperature control. Notably, the majority of the female participants in this trial were postmenopausal, potentially attenuating the biological sex-based protective effects. However, the influence of long-term hormonal exposure cannot be entirely excluded. While our study did not find sex-based differences in outcomes, these underlying biological variations suggest that further research is warranted to explore whether tailored treatment approaches based on sex could optimize outcomes after OHCA.

### Study Limitation

In this study, we did not examine sex differences in prehospital outcomes, but focused solely on a comatose, OHCA population of presumed cardiac course, had achieved ROSC (>20 minutes to 4 hours), and exclusion of unwitnessed asystole, acute stroke, body temperature of <30 °C, and refractory systolic blood pressure <80 mm Hg. Therefore, we cannot rule out the possibility of sex differences in prehospital outcomes. However, among those who survive to hospital admission and receive equal treatment, survival rates appear to be similar between males and females. The study only included OHCA patients with a presumed cardiac cause. Therefore, the findings of this investigation may not be generalizable to in-hospital cardiac arrests or to cases with noncardiac causes.

## Conclusions

Resuscitated males and females who remained comatose upon admission after OHCA demonstrated comparable outcomes when treated with the same protocols for oxygenation, blood pressure, and fever prevention. There were no statistically significant differences in 1-year all-cause mortality rates across the 3 treatment interventions (low compared to high MAP, liberal compared to restrictive oxygenation, or shorter compared to longer duration of temperature control) for both sexes.

Perspectives **COMPETENCY IN MEDICAL KNOWLEDGE:** OHCA remains a critical challenge in emergency medicine, necessitating a profound understanding of the condition and core clinical competencies to improve patient outcomes. Postresuscitation care is important. Notably, female representation in OHCA studies is significantly lower compared to males, resulting in a possible underexamination of treatment effects in females. This randomized study from the BOX trial offers insights into sex-specific outcomes for OHCA. It was found that there is no significant difference in 1-year mortality between males and females or in the efficacy of interventions targeting blood pressure, oxygenation, and temperature control.

**TRANSLATIONAL OUTLOOK:** These findings suggest that medical experts should treat both sexes similarly regarding these specific interventions. Our results indicate that other factors may play a more critical role in determining these patients’ prognosis.

## Funding support and author disclosures

This work received funding from 10.13039/501100009708Novo Nordisk Foundation. The authors have reported that they have no relationships relevant to the contents of this paper to disclose.
