# Supplementary material for: Sex-Specific Outcome Following Targeted Blood Pressure, Oxygenation, and Fever Control After Out-of-Hospital Cardiac Arrest
Source: JACC Adv. 2025 Aug 20;4(10):102056. doi: 10.1016/j.jacadv.2025.102056 (PMC12541223; doi:10.1016/j.jacadv.2025.102056)
Supplement: Supplemental Material [file mmc1.docx]

**Supplemental Table 1.** Baseline characteristics of the males and females allocated to low and high blood pressure targets.

|  | **Female** | | **Male** | |
| --- | --- | --- | --- | --- |
|  | **High MAP  n = 76** | **Low MAP**  **n = 76** | **High MAP**  **n = 317** | **Low MAP**  **n = 320** |
| Age (years), median (IQR) | 63 (53;79) | 64 (50;74) | 64 (56;73) | 65 (53;72) |
| **Comorbidities, n (%)** |  |  |  |  |
| Hypertension | 34 (46) | 41 (54) | 142 (45) | 145 (45) |
| Diabetes | 9 (12) | 10 (13) | 39 (12) | 52 (16) |
| IHD | 13 (17) | 5 (7) | 81 (26) | 73 (23) |
| Chronic heart failure | 10 (13) | 12 (16) | 55 (17) | 60 (19) |
| COPD | 9 (12) | 6 (8) | 21 (7) | 27 (8) |
| Prior stroke | 5 (7) | 8 (11) | 18 (6) | 8 (9) |
| Chronic kidney disease | 6 (8) | 4 (5) | 16 (5) | 13 (4) |
| **Characteristics of the cardiac arrest** |  |  |  |  |
| Shockable rhythm, n (%) | 70 (92) | 63 (83) | 286 (91) | 287 (90) |
| Witnessed arrest, n (%) | 69 (91) | 67 (88) | 270 (85) | 266 (83) |
| Bystander CPR, n (%) | 66 (88) | 61 (81) | 274 (88) | 278 (89) |
| Time to ROSC (minutes), median (IQR) | 15.5 (12.0;29.0) | 20.0 (12.0;25.0) | 20.0 (12.0;26.0) | 17.0 (12.0;25.0) |
| **Findings and procedures at hospital arrival** |  |  |  |  |
| pH, median (IQR) | 7.23 (7.15;7.27) | 7.24 (7.14;7.31) | 7.24 (7.15;7.30) | 7.24 (7.16;7.29) |
| Lactate (mmol/L), median (IQR) | 5.7 (3.8;8.8) | 4.9 (3.0;7.4) | 4.9 (2.6;8.2) | 4.9 (2.9;7.5) |
| paO_2_ (kPa), median (IQR) | 9.3 (6.4;24.9) | 9.9 (6.5;15.7) | 9.9 (6.7;17.9) | 11.5 (7.0;21.5) |
| Temperature (^o^C), median (IQR) | 35.4 (34.4;36.0) | 35.4 (34.2;36.1) | 35.5 (34.8;36.1) | 35.5 (34.8;36.0) |
| ST-segment elevation ECG, n (%) | 32 (42) | 34 (47) | 140 (44) | 144 (47) |
| Immediate coronary angiography, n (%) | 71 (93) | 66 (87) | 293 (92) | 292 (92) |
| Percutaneous coronary intervention, n (%)* | 34 (48) | 29 (44) | 137 (47) | 136 (47) |

*Abbreviations:* MAP: mean arterial pressure; IQR: interquartile range; IHD: ischemic heart disease; COPD: Chronic obstructive pulmonary disease; CPR: cardiopulmonary resuscitation; ROSC: return of spontaneous circulation; SD: standard deviation

* The percentage represents the number of percutaneous coronary interventions performed as a proportion of the total immediate coronary angiographies.

**Supplemental Table 2.** Baseline characteristics of the males and females allocated to restrictive and liberal oxygenation targets.

|  | **Female** | | **Male** | |
| --- | --- | --- | --- | --- |
|  | **Liberal  oxygenation**  **n = 82** | **Restrictive  oxygenation  n = 70** | **Liberal  oxygenation  n = 313** | **Restrictive  oxygenation  n = 324** |
| Age (years), median (IQR) | 65 (55;77) | 62 (50;70) | 65 (55;73) | 63 (54;72) |
| **Comorbidities, n (%)** |  |  |  |  |
| Hypertension | 39 (49) | 36 (51) | 144 (46) | 143 (44) |
| Diabetes | 12 (15) | 7 (10) | 45 (14) | 46 (14) |
| IHD | 10 (12) | 8 (11) | 73 (23) | 81 (25) |
| Chronic heart failure | 16 (20) | 6 (9) | 63 (20) | 52 (16) |
| COPD | 9 (11) | 6 (9) | 25 (8) | 23 (7) |
| Prior stroke | 5 (6) | 8 (11) | 22 (7) | 24 (7) |
| Chronic kidney disease | 5 (6) | 5 (7) | 15 (5) | 14 (4) |
| **Characteristics of the cardiac arrest** |  |  |  |  |
| Shockable rhythm, n (%) | 68 (83) | 65 (93) | 287 (92) | 286 (89) |
| Witnessed arrest, n (%) | 76 (93) | 60 (86) | 263 (84) | 273 (84) |
| Bystander CPR, n (%) | 67 (82) | 60 (88) | 266 (87) | 286 (89) |
| Time to ROSC (minutes), median (IQR) | 20 (12.0;30.0) | 16.5 (12.0;25.0) | 17.0 (12.0;25.0) | 19.0 (12.0;25.0) |
| **Findings and procedures at hospital arrival** |  |  |  |  |
| pH, median (IQR) | 7.23 (7.15;7.30) | 7.24 (7.14;7.27) | 7.25 (7.16;7.30) | 7.23 (7.15;7.29) |
| Lactate (mmol/L), median (IQR) | 5.4 (3.4;8.8) | 4.9 (3.2;7.7) | 4.9 (2.6;8.0) | 4.9 (2.9;7.8) |
| paO_2_ (kPa), median (IQR) | 9.95 (7.22;18.8) | 9.20 (6.00;20.2) | 11.4 (7.00;20.1) | 10.1 (6.70;19.0) |
| Temperature (^o^C), median (IQR) | 35.4 (34.2;36.0) | 35.4 (34.4;36.0) | 35.7 (34.9;36.1) | 35.4 (34.7;36.0) |
| ST-segment elevation ECG, n (%) | 35 (44) | 31 (45) | 136 (44) | 148 (47) |
| Immediate coronary angiography, n (%) | 75 (91) | 62 (89) | 288 (92) | 297 (92) |
| Percutaneous coronary intervention, n (%)* | 32 (43) | 31 (50) | 127 (44) | 146 (49) |

*Abbreviations:* IQR: interquartile range; IHD: ischemic heart disease; COPD: Chronic obstructive pulmonary disease; CPR: cardiopulmonary resuscitation; ROSC: return of spontaneous circulation; SD: standard deviation

* The percentage represents the number of percutaneous coronary interventions performed as a proportion of the total immediate coronary angiographies.

**Supplemental Table 3.** Baseline characteristics of the males and females allocated to fever prevention for 36 hours and 72 hours.

|  | **Female** | | **Male** | |
| --- | --- | --- | --- | --- |
|  | **Temperature control for 36 h n = 73** | **Temperature control for 72 h n = 79** | **Temperature control for 36 h n = 320** | **Temperature control for 72 h n = 317** |
| Age (years), median (IQR) | 63 (54;70) | 65 (49;73) | 64 (54;72) | 64 (55;73) |
| **Comorbidities, n (%)** |  |  |  |  |
| Hypertension | 39 (53) | 36 (47) | 147 (46) | 140 (44) |
| Diabetes | 8 (11) | 11 (14) | 43 (13) | 48 (15) |
| IHD | 9 (13) | 9 (11) | 84 (26) | 70 (22) |
| Chronic heart failure | 9 (13) | 13 (16) | 62 (20) | 53 (17) |
| COPD | 7 (10) | 8 (10) | 19 (6) | 29 (9) |
| Prior stroke | 4 (5) | 9 (11) | 21 (7) | 25 (8) |
| Chronic kidney disease | 3 (4) | 7 (9) | 17 (5) | 12 (4) |
| **Characteristics of the cardiac arrest** |  |  |  |  |
| Shockable rhythm, n (%) | 64 (88) | 69 (87) | 288 (90) | 285 (90) |
| Witnessed arrest, n (%) | 69 (95) | 67 (85) | 269 (84) | 267 (84) |
| Bystander CPR, n (%) | 61 (85) | 66 (85) | 279 (89) | 273 (87) |
| Time to ROSC (minutes), median (IQR) | 17.0 (11.0;25.0) | 20.0 (13.5;28.0) | 19.0 (12.0;26.0) | 17.0 (12.0;25.0) |
| **Findings and procedures at hospital arrival** |  |  |  |  |
| pH, median (IQR) | 7.23 (7.17;7.28) | 7.24 (7.11;7.27) | 7.23 (7.14;7.29) | 7.25 (7.17;7.30) |
| Lactate (mmol/L), median (IQR) | 4.7 (3.4;7.1) | 5.7 (3.3;8.7) | 5.3 (3.0;8.1) | 4.6 (2.4;7.7) |
| paO_2_ (kPa), median (IQR) | 9.15 (6.70;23.5) | 9.80 (6.50;17.7) | 11.3 (7.2;19.9) | 10.6 (6.30;18.9) |
| Temperature (^o^C), median (IQR) | 35.7 (34.3;36.2) | 35.3 (34.4;35.9) | 35.6 (34.9;36.1) | 35.5 (34.6;36.1) |
| ST-segment elevation ECG, n (%) | 31 (43) | 35 (46) | 141 (45) | 143 (46) |
| Immediate coronary angiography, n (%) | 66 (90) | 71 (90) | 293 (92) | 292 (92) |
| Percutaneous coronary intervention, n (%)* | 34 (52) | 29 (41) | 127 (43) | 146 (50) |

*Abbreviations:* IQR: interquartile range; IHD: ischemic heart disease; COPD: Chronic obstructive pulmonary disease; CPR: cardiopulmonary resuscitation; ROSC: return of spontaneous circulation; SD: standard deviation

* The percentage represents the number of percutaneous coronary interventions performed as a proportion of the total immediate coronary angiographies.

**Supplemental Table 4**. Cox proportional hazard models for 1-year all-cause mortality adjusted for admission site.

| Cox proportional hazard model (1-year all-cause mortality) | | | |
| --- | --- | --- | --- |
| Intervention strategies* | **HR** | **95% CI** | **p** |
| Blood pressure, female (HR with low) | 1.00 | (0.61-1.64) | 0.998 |
| Blood pressure, male (HR with low) | 0.89 | (0.69-1.16) | 0.403 |
| Oxygenation, female (HR with restrictive) | 0.81 | (0.49-1.35) | 0.425 |
| Oxygenation, male (HR with restrictive) | 0.96 | (0.74-1.25) | 0.754 |
| Temperature control, female (HR with 36 hours) | 0.73 | (0.44-1.21) | 0.226 |
| Temperature control, male (HR with 36 hours) | 1.08 | (0.83-1.40) | 0.592 |

*Abbreviations:* HR: hazard ratio; CI: confidence interval.

*Comparison of intervention strategies: For the blood pressure intervention, a target mean arterial pressure of 63 mm Hg was compared to 77 mm Hg for both sexes. For the oxygenation intervention, an arterial oxygen concentration range of 9–10 kPa was compared to 13–14 kPa for both sexes. For the temperature control intervention, device-based fever prevention was implemented for 36 hours and compared to 72 hours for both sexes.
